# Wall Shear Stress Estimation of Thoracic Aortic Aneurysm Using Computational Fluid Dynamics

**DOI:** 10.1155/2018/7126532

**Published:** 2018-06-03

**Authors:** J. Febina, Mohamed Yacin Sikkandar, N. M. Sudharsan

**Affiliations:** ^1^Department of Biomedical Engineering, GRT Institute of Engineering and Technology, Tiruttani, India; ^2^Department of Medical Equipment Technology, College of Applied Medical Sciences, Majmaah University, Al Majmaah 11952, Saudi Arabia; ^3^Department of Mechanical Engineering, Rajalakshmi Engineering College, Chennai, India

## Abstract

An attempt has been made to evaluate the effects of wall shear stress (WSS) on thoracic aortic aneurysm (TAA) using Computational Fluid Dynamics (CFD). Aneurysm is an excessive localized swelling of the arterial wall due to many physiological factors and it may rupture causing shock or sudden death. The existing imaging modalities such as MRI and CT assist in the visualization of anomalies in internal organs. However, the expected dynamic behaviour of arterial bulge under stressed condition can only be effectively evaluated through mathematical modelling. In this work, a 3D aneurysm model is reconstructed from the CT scan slices and eventually the model is imported to Star CCM+ (Siemens, USA) for intensive CFD analysis. The domain is discretized using polyhedral mesh with prism layers to capture the weakening boundary more accurately. When there is flow reversal in TAA as seen in the velocity vector plot, there is a chance of cell damage causing clots. This is because of the shear created in the system due to the flow pattern. It is observed from the proposed mathematical modelling that the deteriorating WSS is an indicator for possible rupture and its value oscillates over a cardiac cycle as well as over different stress conditions. In this model, the vortex formation pattern and flow reversals are also captured. The non-Newtonian model, including a pulsatile flow instead of a steady average flow, does not overpredict the WSS (15.29 Pa compared to 16 Pa for the Newtonian model). Although in a cycle the flow behaviour is laminar-turbulent-laminar (LTL), utilizing the non-Newtonian model along with LTL model also overpredicted the WSS with a value of 20.1 Pa. The numerical study presented here provides good insight of TAA using a systematic approach to numerical modelling and analysis.

## 1. Introduction

Aorta is the major artery that carries blood from heart to all parts of the body and part of the aorta that runs through the chest is called thoracic aorta [1]. When an area of thoracic aorta expands or bulges, it is called a thoracic aortic aneurysm (TAA). According to the study of incidence and mortality rate of TAA, the incidence of ruptured thoracic aneurysms in individuals aged 60–69 years is about 100 cases per 10,000; in those aged 70–79 years, it is about 300 cases per 10,000 and in those aged 80–89 years, it is about 550 cases per 10,000 people [1]. TAA causes plaque formation, which is a serious health risk as it can burst or rupture the inner wall intima continuing to the outer wall adventitia. However, plaque cap rupture per se does not lead to stoppage of blood flow. Rather, plaque cap rupture provides a surface that initiates thrombosis, which may then grow to occlude the vessel and stop blood flow [2]. TAA exists in both saccular shape and fusiform shape. The saccular shape aneurysm is eccentric, involving only one portion of the circumference of an aortic wall. The fusiform aneurysm is concentric and involves the full circumference of the vessel wall. The effect of high shear hemodynamics on thrombus growth has profound implications for the understanding of all acute thrombotic cardiovascular events as well as for vascular reconstructive techniques and vascular device design, testing, and clinical performance [3]. In the present work, saccular shape TAA is considered in detail due to the fact that it has caused greater rupture risks than fusiform aneurysm [3]. This work aims to show that Computational Fluid Dynamics (CFD) is an effective tool that can provide better insights into TAA diagnosis with proper solver settings and a numerical protocol.

The viscosity values for a group of five normal people were estimated by Stein et al. [4] to be in the range of 0.0051 to 0.0055 Ps s (average value of 0.0053 Pa s) and the same has been used in the present study (Table 1). The aorta diameter was computed using the peak Reynolds number, peak velocity, and viscosity. This value was then used to obtain the average velocity and Reynolds number. The density value of blood is taken to be *ρ* = 1060 kg/m^3^. It can be seen from table that the flow of blood inside aorta is predominantly laminar in nature. Owing to the pulsatile nature, the flow is sometimes (locally) turbulent. The derived values obtained from Stein et al. [4] are shown in Table 1.

From Table 1, it is clear that turbulent flow occurs only with peak blood flow velocity values. With average velocity values, the flow tends to be laminar with the average Reynolds number in the range of 730 to 856. A regular geometry profile that mimics an aneurysm was modelled and numerically solved by Berguer et al. [5]. Both laminar and turbulent pulsatile flows were studied. The actual diseased model was not studied. The effects of Newtonian and non-Newtonian behaviours of blood were compared. They concluded that non-Newtonian turbulent flow is to be considered in the study of aneurysms. It is to be noted that the pulsatile flow will be both laminar and turbulent based on the velocity attained at that instant of time. A transit model that could best handle both laminar and turbulent flow would be a better predictor of the flow phenomenon and provide more acceptable wall shear stress values.

A transitional flow model based on Menter et al. [6] was employed by Tan et al. [7] for predicting the blood flow patterns in a fusiform aneurysm region assuming the blood to be Newtonian in nature. In essence, although the flow was pulsatile with a major pulse component being laminar in nature, the transitional flow model solves the fluid flow in the near wall region as laminar and turbulent elsewhere. From the simulation results, they observed that the transitional model gave better inference compared with the laminar simulation. Highly disturbed, recirculating flow was observed within the bulged region of the aneurysm. High turbulence intensity values were particularly observed near the outlet of the aneurysm. It was also presented that the wall shear stress values obtained could be an overestimate. It would be of interest to investigate an appropriate transitional model considering blood to be non-Newtonian in nature on a saccular aneurysm of a diseased aorta. However, the effective implementation of this basic scheme requires a very fine grid resolution near the wall region. The suggested y^+^ (nondimensional distance from wall) value is < 2. This is extremely fine and would be computationally intensive. The detailed significance and explanation of computation is presented under the numerical protocol section of this paper. An optimum solution to this would be to achieve a good prediction even with a comparative fine mesh having a selected wall y^+^ value of 100, at the same time taking care to ensure that the laminar/turbulent transition is in a pulsatile flow instead of using the typical transitional flow model.

A. C. Benim et al. [8] observed that, for a normal human aorta, time averaged velocity field of pulsatile flow did not show remarkable differences in steady-state results. They indicated that the mobilization of atherosclerotic plaques needs to be considered as a very important issue for extracorporeal circulation. However, the effect of actual flow pattern on a diseased aorta was, however, not studied. A detailed lumen surface representation of aortic aneurysm is very important in the analysis of stress pattern [9]. However, the fluctuations of blood flow inside an aneurysm lumen region were not studied in detail. Qiao et al. described the formation and development of aortic plaque in an aneurysm region using CFD [10]. The simulation was performed for a fusiform aneurysm in the descending aorta with pulsatile flow but the cell refinement and grid independence test were not performed. Callaghan et al. presented a work that combined a 4D flow and CFD simulation of a thoracic aortic aneurysm case [11]. They reported that high wall shear stress values of 20 Pa were found in the ascending aorta during the turbulent flow and concluded that the CFD provides results supplementing the 4D flow data in the understanding of aneurysm development and risk; however, the laminar flow and significance of wall y+ in the aneurysm model were not studied. Numata et al. [12] and Markl et al. [13] reported that CFD simulation alone does not guarantee fidelity to reproduce in vivo hemodynamics due to inherent model limitations. They noted that the grid resolution errors were a possible source of uncertainty. In order to overcome this, mesh independence study was performed. Soudah et al. [14] provided a detailed methodology and the importance of wall y^+^ for capturing the WSS. However, it was computed for the peak systolic instant time using flat inlet velocity profile for a normal aorta. Basri et al. [15] detailed the recent usage of CFD in biomedical applications. The authors reviewed several research papers that studied the use of CFD as a tool for determining the pathophysiology of a cardiovascular system. The abnormalities discussed by the authors included narrowing of aortic wall, leakage of blood from the valve opening, and stenosis conditions. However, the usage of CFD in aneurysm condition was not studied.

In summary, from the analysis of available literature, it is seen that it is necessary to consider a geometry model that is as close as possible to the real aorta. This can be ensured by using one of the several imaging modalities, like CT. This model has to be discretized (meshed) with proper refinement close to the wall and must be fine enough to avoid numerical errors. In this research work, an aorta having a saccular aneurysm is reconstructed from a CT image. The detailed discretization scheme is an extension of [14] with improvements in mesh topology which are explained in detail in the next section. As far as the physics is concerned, it is seen from literature that the flow should be considered as pulsatile and fluid (blood) as non-Newtonian. The discretization of the geometry is very important to obtain a solution with minimum numerical error. The protocol adopted is presented in detail in the next section. The numerical scheme is the key to accurate prediction. From the study of available literature, it is seen/noted that several schemes have been tried, namely, laminar, turbulent, and transitional models assuming a steady velocity profile and/or pulsatile profile with a Newtonian behaviour. A pulsatile flow with non-Newtonian fluid model has not been attempted thus far/yet and it is suggested that this improved method of approach with above-mentioned modelling parameters would provide a more accurate estimation of WSS values.

In this research work, an attempt is made to test the efficacy of the numerical scheme by comparing the results obtained using a pulsatile velocity profile for both Newtonian and non-Newtonian models considering the flow to be laminar. The reason for using the laminar flow model is that for a pulse of 0.8 seconds the flow is turbulent only from 0.02 to 0.2 seconds, where the Re values exceed 2200. The average Re is always less than 2200. To find an alternate to the transitional model that assumes the bulk of the fluid transport as fully turbulent (which it is not) and also requires a very fine mesh (a computationally intensive proposition), it is proposed to use a laminar-turbulent switch based on the Reynolds number. This is to see if the possibility of overprediction of WSS [8] can be mitigated. The velocity trace can be mapped to a laminar-turbulent-laminar flow from a cardiac cycle. In this model, the velocity trace is mapped to the Reynolds number. For all values of Re < 2200, the laminar flow solver is enabled, and for Re > 2200, the standard k-epsilon model is enabled.

## 2. Materials And Methods

### 2.1. Numerical Evaluation Protocol

**Figure 1:**
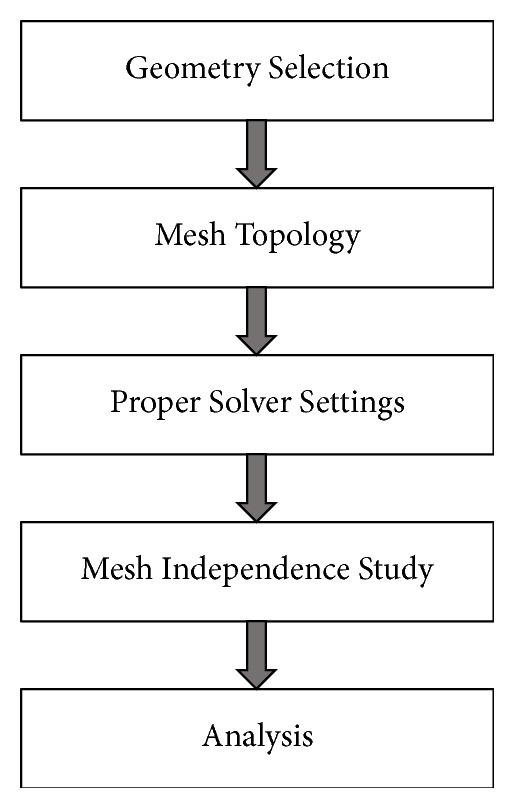
Workflow.

The workflow followed in this study is pictorially represented in Figure 1 and is briefly explained below.

#### 2.1.1. Geometry Selection

In this research, the 3D aneurysm model was reconstructed from the CT scan slices using MIMICS. The original CT image file format was DICOM. The total number of scanning slices is 600 and the range of scanning was from neck to legs. The distance between neighbouring layers was 1 mm. The CT data was imported into MIMICS software and data of the aortic vessel was extracted by means of 3D threshold segmentation. Then, the model is imported to Star CCM+ (Siemens, USA) for CFD analysis. The domain is discretized using polyhedral mesh with prism layers to capture the boundary more accurately based on the following reasons.

#### 2.1.2. Polyhedral Mesh

Polyhedral meshes provide a balanced solution for complex mesh generation problems. They are relatively easy and efficient to build, requiring no more surface preparation than the equivalent tetrahedral mesh. They also contain approximately five times fewer cells than a tetrahedral mesh for a given starting surface. Multiregion meshes with a conformal mesh interface are allowed.

#### 2.1.3. Prism Layer Mesh

**Figure 2:**
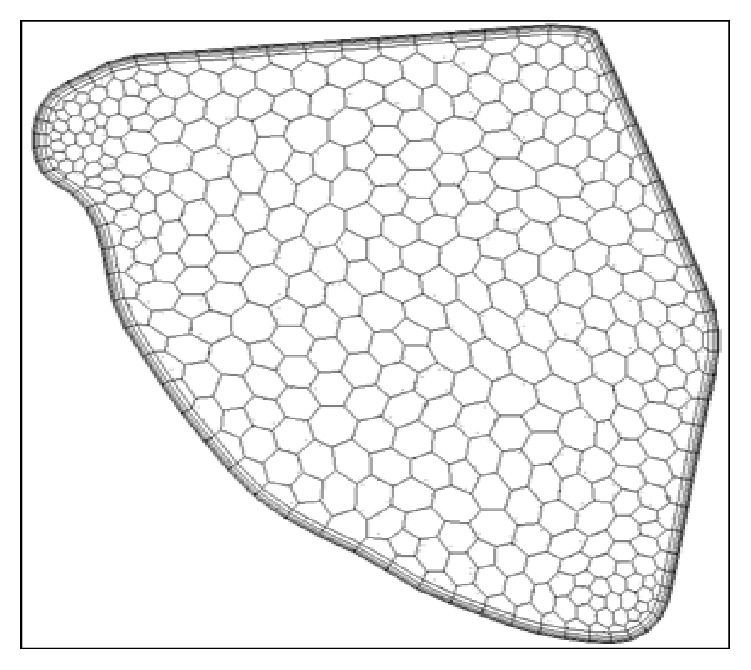
Prism layer at the outlet.

The prism layer mesh model as shown in Figure 2 is used with a core volume mesh to generate orthogonal prismatic cells next to wall surfaces or boundaries. This layer of cells is necessary to improve the accuracy of the flow solution and provides a conformal mesh for the model. This helps in capturing the velocity across the boundary layer more accurately than depending on standard wall functions. This is decided from the wall y+ value that is discussed later in this section. It is to be noted that Figure 2 presents only the representation of the prismatic layer near wall with polyhedral cells in the center. The actual mesh is extremely fine (average distance from the wall to the adjacent grid is 0.0001 m.) and one would not be able to see the geometric progression of cell layer thickness from wall to the inner fluid region.

#### 2.1.4. Significance of Wall y+ Value

To understand the actual physics of wall shear stress, the flow close to the boundary layer has to be accurately captured. Since the boundary layer thickness is extremely small, the first grid point must be very close to the wall. This distance from the wall is represented as nondimensionless wall y^+^.

(1) $$\begin{array}{c} y^{+}=\frac{yu_{\tau }}{\vartheta },\;u_{\tau }=\sqrt{\frac{\tau_{w}}{\rho }} \end{array}$$

where

  y^+^ is nondimensional distance from the wall,
  y is the distance from the wall, m,
 
*u*_*τ*_ is frictional velocity,
 
*ϑ* is kinematic viscosity, m/s^2^,
 
*τ*_*w*_ is wall shear stress, Pa,
 
*ρ* is density, kg/m^3^.

**Figure 3:**
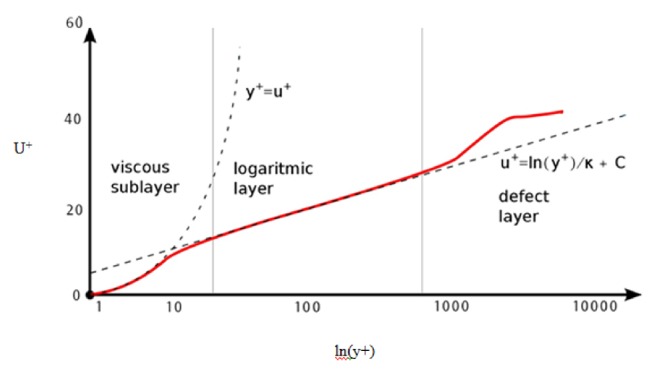
Nondimensional distance from wall y+ versus nondimensional velocity U+.

The velocity profile close to the wall is generally assumed to be parabolic and the boundary layer variation is assumed to be parabolic. However, this is not the case. The nondimensional velocity u+ is plotted against the nondimensional distance from the wall and defined as y+ (Figure 3). As seen in Figure 3, the boundary layer can be divided into three regions and they are viscous sublayer, log layer, and defect layer. In the viscous sublayer y+ = u+ and this holds good for a value of y+ up to 5. It means that the viscous forces are as strong as the inertial forces. In the log layer, the u+ value increases exponentially in comparison to the distance from the wall and is linear when plotted in logarithmic scale.

Wall y+ value indicates the position of the grid inside the boundary layer. A value of less than five signifies that the first grid is in viscous sublayer and a value of 300 signifies that the first grid is in log layer. It is desirable that y+ be in this range to ensure that the physics of boundary layer are truly represented in this study's computation even though standard wall functions are enabled. In this case, y+ ranges from 3 to 100, respectively. This clearly satisfies the condition that, in the present simulation, the flow inside boundary layer is fully represented by the prism layer grid points.

#### 2.1.5. Mesh Independence Study

**Figure 4:**
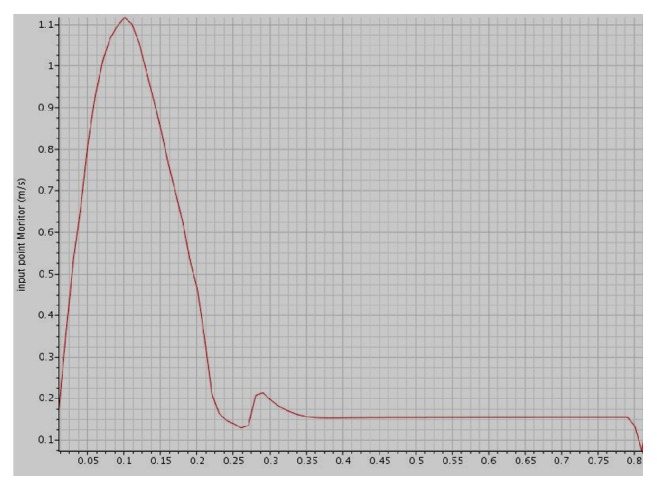
Velocity profile graph [15].

In order to avoid the grid resolution errors, three different mesh models are generated in the fluid domain. The number of cells generated is two, four, and eight hundred thousand, respectively. The number of prism layers is set to four for capturing the near-wall velocity profile. The simulations were performed for pulsatile velocity profile of a typical resting condition on the aneurysm region in thoracic aorta and the results presented are of a male person (age 61) in Figure 4. The velocity profile was obtained from the published literature [15].

**Figure 5:**
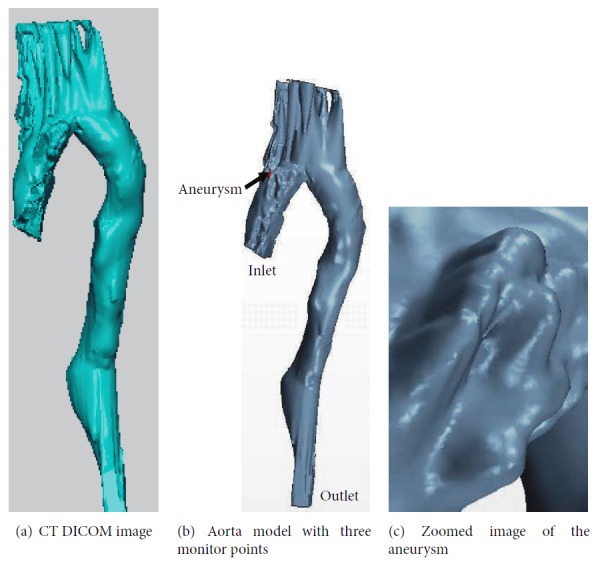


**Figure 6:**
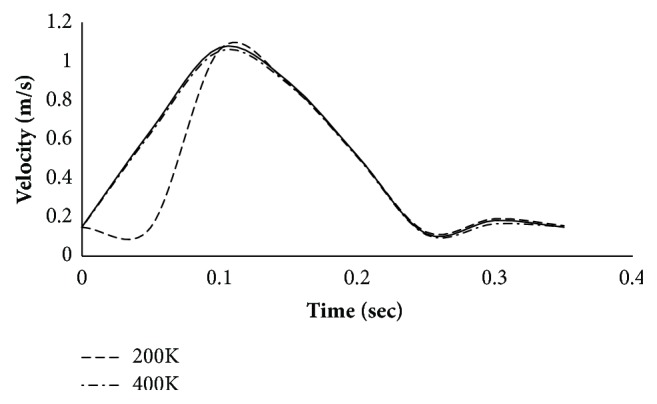
Velocity profile over time at monitoring point 1 (inlet) for various mesh counts.

**Figure 7:**
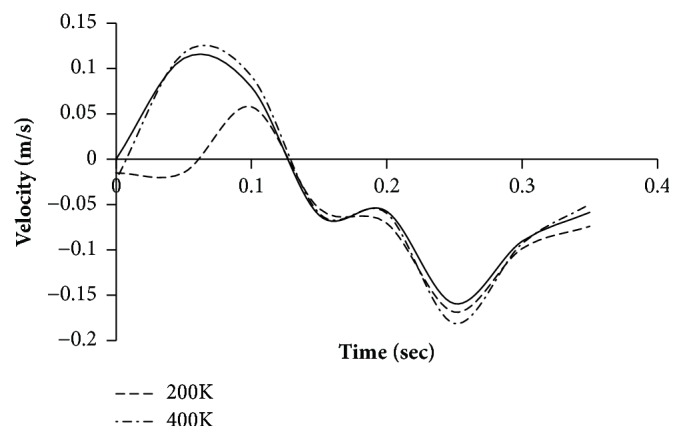
Velocity profile over time at monitoring point 2 (aneurysm) for various mesh counts.

**Figure 8:**
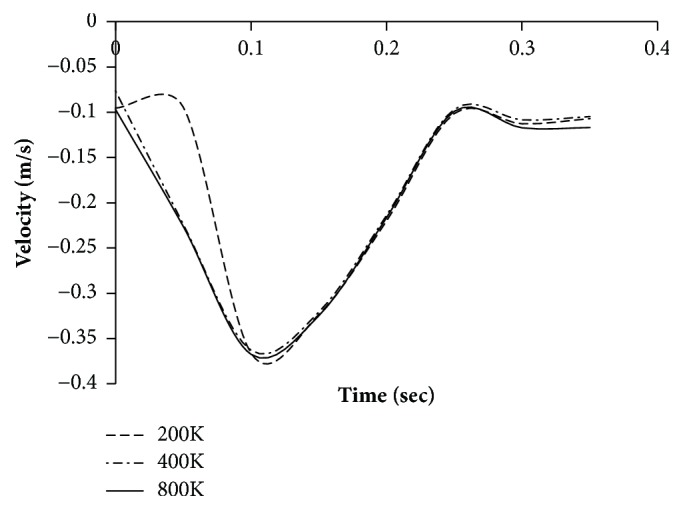
Velocity profile over time at monitoring point 3 (outlet) for various mesh counts.

The simulation is performed assuming Newtonian behaviour. The simulation is run for 6 cycles to allow the solver to stabilize and the results for the seventh cycle are plotted. Three points are monitored. Point 1 corresponds to inlet, point 2 corresponds to the location where the aneurysm is present, and point 3 refers to the outlet (Figure 5). Figures 6–8 present the velocity profile over time for various mesh counts. The negative value in the velocity is because the flow is considered to be positive in the upward direction in the inlet region. It can be seen from all the three figures that the mesh-independent solution is reached for a mesh count of 400,000. However, for ensuring fidelity, all further simulations are performed with a refined mesh having a mesh count of 800,000.

#### 2.1.6. Solver Settings

Regarding physical conditions, they include unsteady, pulsatile, and laminar, segregated flows. The laminar flow is considered based on the value obtained from the Reynolds number. The average Reynolds number for pulsatile flow is 1405. From the velocity profile, it is found that 78% is laminar and 22% is turbulent. As the flow is predominantly laminar, the laminar flow model is chosen. However, the laminar-turbulent-laminar (LTL) model is also simulated for comparison.

Regarding boundary conditions, the inlet boundary condition is velocity inlet and the velocity profile given in the inlet is shown in Figure 5(a). For CFD analysis, the average velocity has to be set as an inlet boundary condition instead of using peak velocity value. The wall is assumed to be rigid and no slip condition is set in the wall. The outlet boundary condition is the pressure outlet.

For Newtonian or non-Newtonian fluid, generally, blood follows a Newtonian behaviour in large arteries and non-Newtonian behaviour in small arteries and capillaries. But in both medium and large sized blood vessels, non-Newtonian behaviour influences hemodynamic factors [16–18]. To understand this effect, non-Newtonian model of blood is also adopted and compared with the results obtained using Newtonian flow. The viscosity of the blood is set at 0.0052 Pa-s based on the experimental results reported in [4].

**Figure 9:**
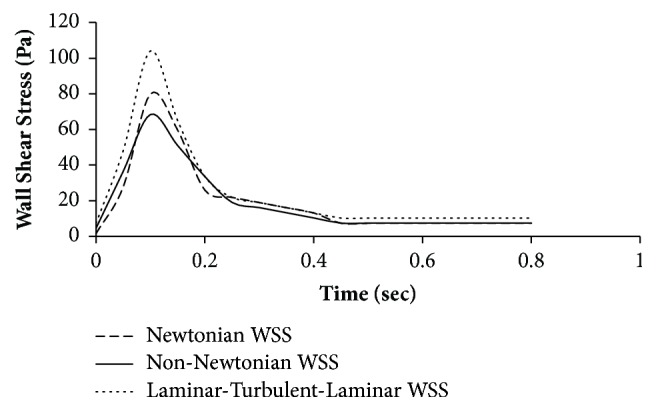
Pulsatile flow wall shear stress versus time for Newtonian and non-Newtonian WSS.

During the steady state, non-Newtonian behaviour affects the wall shear stress predicting larger values than the Newtonian model [16, 17, 19–21]. Solving this aorta model assuming a steady average velocity, WSS value is estimated as 13.2 Pa for non-Newtonian model, which is higher than the WSS value of Newtonian model (7.4 Pa). But when pulsatile flow is considered, Newtonian behaviour tends to provide larger WSS than the non-Newtonian behaviour as can be seen from the graph presented above (Figure 9) with average values of 16 Pa and 15.29 Pa, respectively. The graph also shows the laminar-turbulent-laminar (LTL) model proposed in this paper. This model (LTL) overpredicts WSS with an average value of 20.10 Pa. In effect, it is amply evident from this work and others that considering blood flow as laminar is the right assumption/presumption.

**Figure 10:**
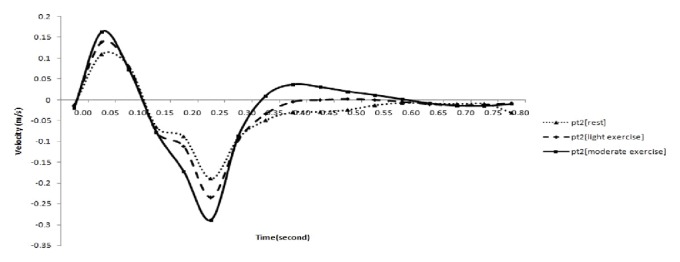
Velocity in the aneurysm region at different exercise conditions. Note: pt2 indicates the point in the aneurysm region.

The simulation was performed based on three conditions: during rest, light exercise, and moderate exercise. The velocity profile varies with the stress condition and the same is presented in Figure 10.

## 3. Result and Discussion

We consider the velocity profile and vorticity at the three peaks at 0.05, 0.25, and 0.75 time instants in the velocity profile of blood flow during rest, light exercise, and moderate exercise positions (Figure 10).

From Figure 10, it is observed that, for moderate exercise conditions, the velocity profile has peaks at 0.05, 0.25, 0.4, and 0.75 seconds and two peaks for the other two stress conditions. The corresponding velocity vector at these three time instants is plotted in Figure 10.

**Figure 11:**
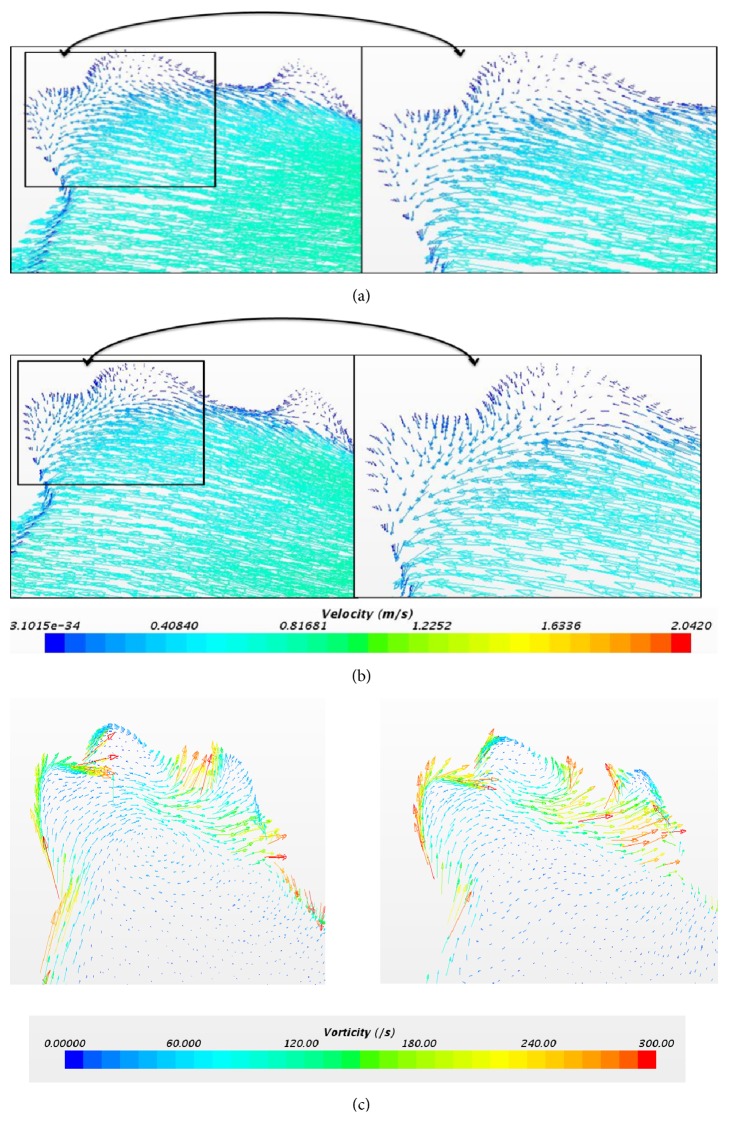
(a) Velocity profile at 0.05 sec using laminar method. (b) Velocity profile at 0.05 sec using laminar-turbulent-laminar scheme. (c) Vorticity profile using laminar and laminar-turbulent-laminar methods at 0.05 sec.

Figures 11(a) and 11(b) indicate the vector scene represented during the first peak in Figure 10 at the aneurysm point for both laminar and laminar-turbulent-laminar methods. This shows that the flow is towards the left side, which makes the base for the formation of vortex.  Figure 11(c) presents the vorticity plot at the same time instant. The vorticity value at the aneurysm using laminar method is 110.7285(/s) and for LTL method it is 221.6708 (/s). Although the pulsatile flow behaves as a laminar-turbulent-laminar based on the velocity profile, it overpredicts the values when compared to a laminar method. This again fortifies that the simulation needs to be performed using a non-Newtonian and laminar scheme.

**Figure 12:**
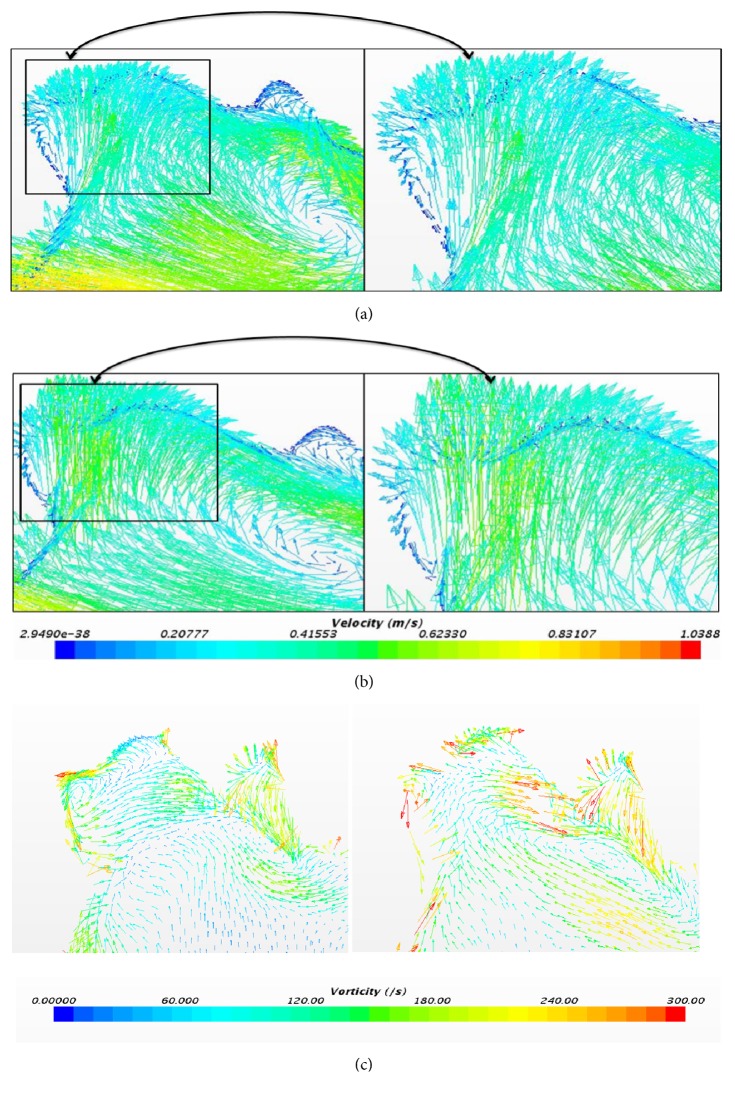
(a) Velocity profile at 0.25 sec using laminar method. (b) Velocity profile at 0.25 sec using laminar-turbulent-laminar scheme. (c) Vorticity profile using laminar and laminar-turbulent-laminar methods at 0.25 sec.

Figure 12 indicates the flow reversal in the aneurysm region (i.e., flow towards the right).  Figure 12(c) presents the vorticity plot at the same time instant. The vorticity value at the aneurysm using laminar method is 111.07(/s) and for LTL method it is 182.44 (/s).

**Figure 13:**
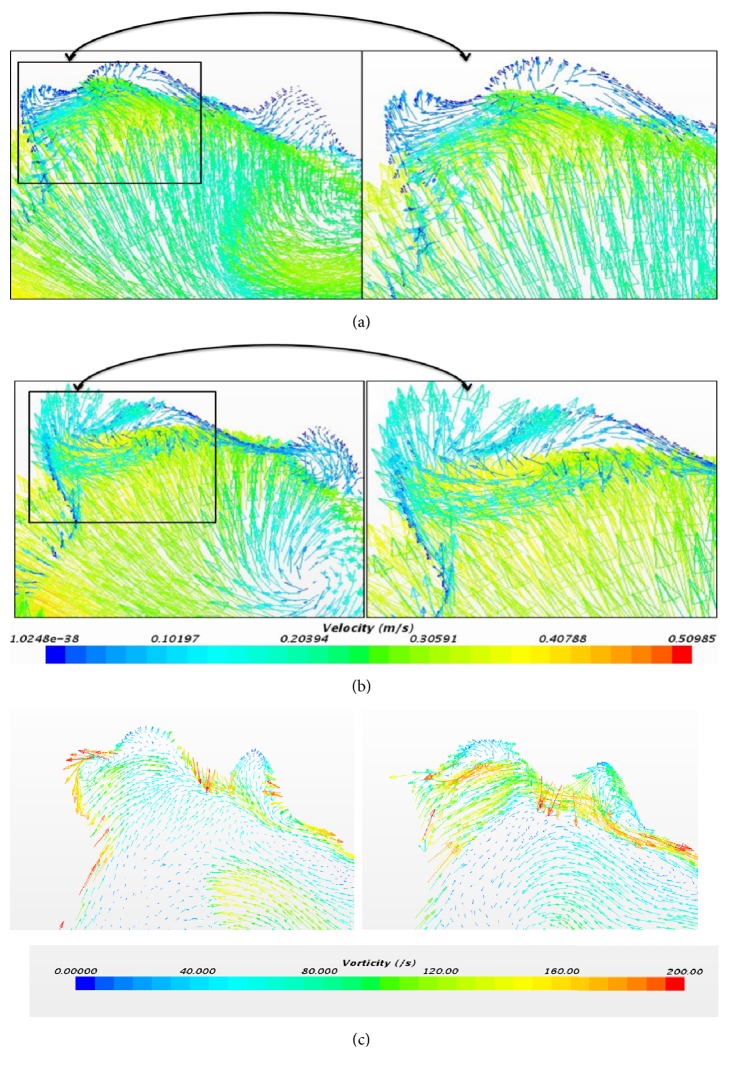
(a) Velocity profile at 0.4 sec using laminar method. (b) Velocity profile at 0.4 sec using laminar-turbulent-laminar scheme. (c) Vorticity profile using laminar and laminar-turbulent-laminar method at 0.4 sec.

In Figure 13, the flow is towards the left side and this vector scene is obtained during the second positive peak in Figure 10.  Figure 13(c) presents the vorticity plot at the same time instant. The vorticity value at the aneurysm using laminar method is 19.39(/s) and for laminar-turbulent-laminar method it is 46.98 (/s).

**Figure 14:**
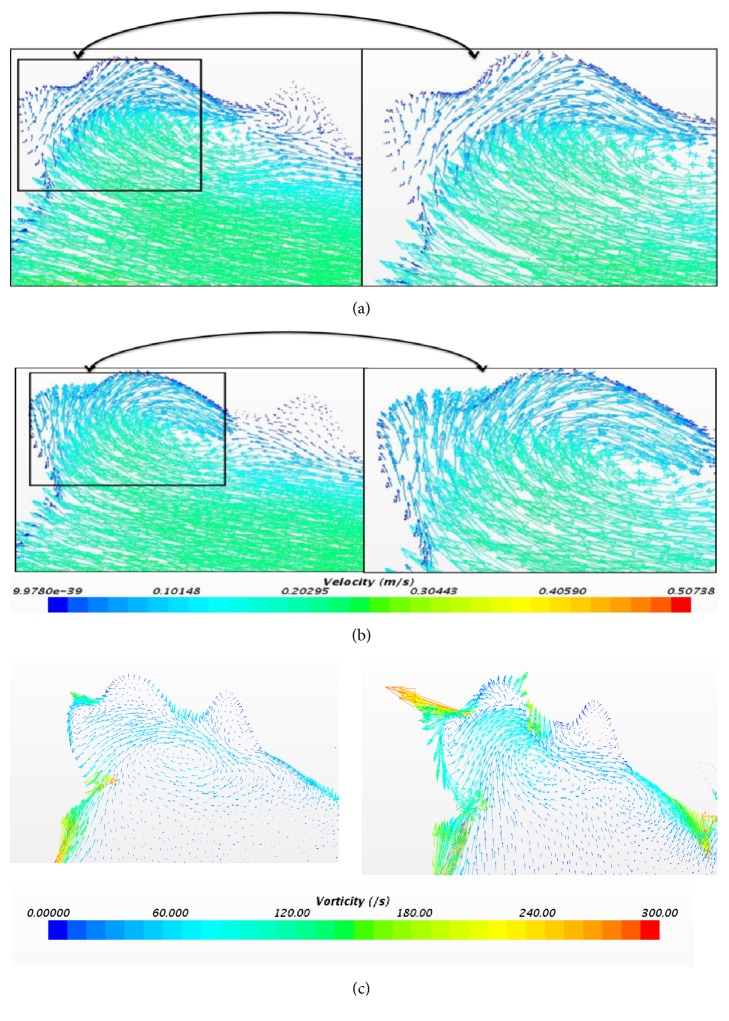
(a) Velocity profile at 0.75 sec using laminar method. (b) Velocity profile at 0.75 sec using laminar-turbulent-laminar scheme. (c) Vorticity profile using laminar and laminar-turbulent-laminar method at 0.75 sec.

In Figures 14(a) and 14(b), the flow vector direction is towards the right, indicating the flow reversal in the aneurysm region again. This flow reversal may have an effect on wall abrasion and the possibility of rupture. There is an increase in peak velocity of flow in the ascending aorta with exercise [22].  Figure 14(c) presents the vorticity plot at the same time instant. The vorticity value at the aneurysm using laminar method is 13.5542 (/s) and for laminar-turbulent-laminar method it is 31.3758(/s).

According to Taylor [23], for a normal person, reverse flow occurs in aorta during rest and reverse flow gets eliminated during exercise. The present model was carried out on subject with aneurysm and from the analysis it was found that there is reverse flow and flow fluctuations during exercise condition in the aneurysm region, which could lead to rupture.

Although the LTL scheme provides the same vector scene as non-Newtonian model, the WSS is comparatively high for the laminar-turbulent-laminar scheme. Also the vorticity value is high for the laminar-turbulent-laminar scheme when compared to the laminar scheme.

**Figure 15:**
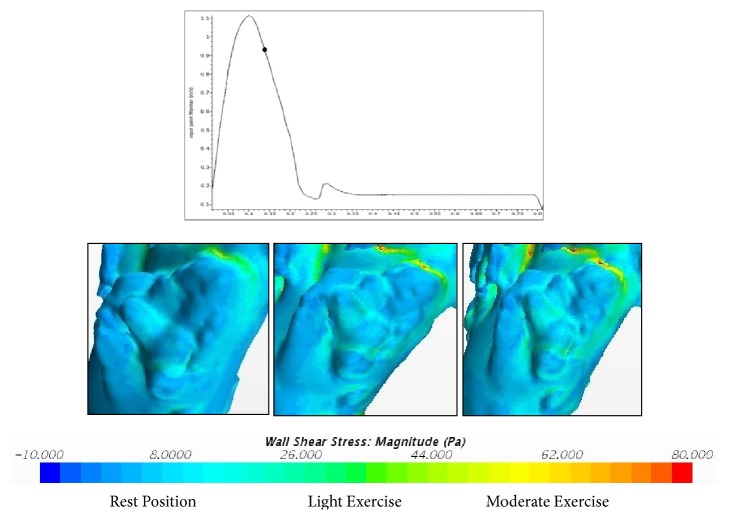
WSS during rest, light exercise, and moderate exercise at 0.15 sec.

**Figure 16:**
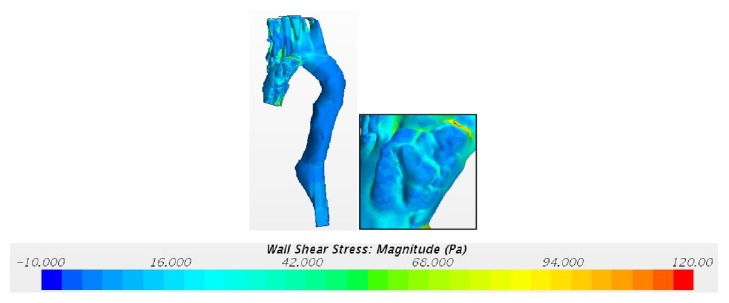
Wall shear stress during moderate exercise using the laminar-turbulent-laminar scheme.

The WSS is associated with blood flow through an artery and also depends on the size and geometry of an aorta. The corresponding WSS during rest, light exercise, and moderate exercise are shown in Figures 15 and 16.

From this, it is clear that laminar-turbulent-laminar scheme overpredicts high WSS values. So, the alternate choice of the non-Newtonian model with laminar flow can provide better results among the different models discussed above.

WSS is an important factor for rupture and plaque formation. Results so far published in open literature have emphasised that the WSS is overpredicted in a numerical experiment. Numerical results heavily depend on the settings and these have not been well documented so far. In order to obtain solutions that match the physical experiments, there is a need for (a) proper extraction of the geometry without loss of detail and a good discretization scheme; (b) choice of flow; and (c) choice of physics. These are explained in detail below.

The discretization of the geometry must follow a protocol in such a way that the near-wall region is adequately captured along with a mesh-independent study to check the fidelity of the solution. Although the flow behaviour is laminar-turbulent-laminar over a cardiac cycle, it has been seen that this model overpredicts WSS as well as the vorticity. Surprisingly, it is assumed that the flow to be laminar provides a better estimate for WSS. Also the physics of the fluid are taken as non-Newtonian and this provides a better result than Newtonian. It is to be noted that a pulsatile flow with non-Newtonian fluid model has not been attempted so far and seen as an improved method of approach with above-mentioned modelling parameters to estimate the WSS more accurately.

Also, the development of vortex and flow reversal is presented in this work, which is a possible reason for formation of plaque deposition. This is a cause for reduction in flow area and increase in internal pressure. This insight on the flow reversal and vortex development has not yet been explored based on the available literature to the best of the authors' knowledge. Chatzizisis et al. and Chen et al. have presented that, at locations where there is a presence of atherosclerotic lesions, oscillatory or low shear stress is predominantly observed [24, 25]. However, Chen et al. also observe that effect of stress on plaque is unknown [25].

## 4. Conclusion

The conformal mesh for the model is obtained using the polyhedral and prism layer mesh. The flow along the sides of the wall is captured well by using wall y+. It has been observed that considering pulsatile blood flow to be laminar is correct and it does not overpredict the WSS values. Moreover, it is also found necessary to consider the blood to be non-Newtonian even when the flow is in the larger blood vessel. The true representation of the geometry, discretization with fine meshes in the near wall region (y^+^ < 100), and the proper choice of numerical scheme are the keys to correct prediction of flow and WSS. CFD as a mathematical tool can help in understanding the flow physics phenomena inside an artery. The advantage is that the model can be tested for varying stresses that an artery may be subjected (to) in day to day life. This tool will help in evaluating treatment methodology and suggest lifestyle modifications for the diseased patient. Thus, the relationship between CFD and cardiovascular hemodynamics was studied with different assumptions. It was found that CFD can provide better results with proper modelling, mesh, and solver settings. Limitation of this study is that the present findings are based on limited population (i.e., one particular case). In future, more CT data of saccular shape thoracic aortic aneurysm patients can be obtained and then simulation can be done to provide more appropriate numerical results.

## Figures and Tables

**Table 1:** Derived values of aortic diameter, average velocity, and Reynolds number.

	D = (Re*∗µ*)/(Vpeak)	Vavg = Q/A	Reavg = (*ρ∗*D*∗*Vavg)/*µ*
**Patient No.** ^*∗*^	**Aortic diameter (D) (m)**	**Average velocity (m/sec) (V avg)**	**Average Reynolds number (Re avg)**
2	0.0302	0.1229	730.03

3	0.0287	0.1333	797.38

4	0.0262	0.1629	856.39

5	0.0261	0.1674	844.23

6	0.0304	0.1281	811.12

^*∗*^Table 1 of Stein et al. [4].

## Data Availability

The clinical data are available upon request in accordance with the volunteers' informed consent. The data will not be shared online.
